# Research on Spatial Optical Path System for Evaluating the Reflection Performance of Quartz-Based Volume Bragg Grating Applied to Fabry–Perot Cavity

**DOI:** 10.3390/mi16090998

**Published:** 2025-08-29

**Authors:** Jiamin Chen, Gengchen Zhang, Hejin Wang, Qianyu Ren, Yongqiu Zheng, Chenyang Xue

**Affiliations:** State Key Laboratory of Extreme Environment Optoelectronic Dynamic Measurement Technology and Instrument, North University of China, Taiyuan 030051, China; cjiamin@nuc.edu.cn (J.C.); sz202306191@st.nuc.edu.cn (G.Z.); wanghejin23@163.com (H.W.); renqianyu@nuc.edu.cn (Q.R.); xuechenyang@nuc.edu.cn (C.X.)

**Keywords:** volume Bragg grating, fused quartz, reflectivity measurement, space optical path, air gap Fabry–Perot cavity

## Abstract

In the field of high-temperature in situ sensing, highly reflective Fabry–Perot (F-P) cavity mirrors with thermal stress matching are urgently needed. The quartz-based volume Bragg grating (VBG) can replace the dielectric high-reflection film to prepare a high-temperature and high-precision F-P cavity sensitive unit by virtue of the integrated structure of homogeneous materials. The reflectivity of the VBG is a key parameter determining the performance of the F-P cavity, and its accurate measurement is very important for the pre-evaluation of the device’s sensing ability. Based on the reflectivity measurement of quartz-based VBG with a large aspect ratio, a free-space optical path reflective measurement system is proposed. The ZEMAX simulation is used to optimize the optical transmission path and determine the position of each component when the optimal spot size is achieved. After completing the construction of the VBG reflectivity measurement system, the measurement error is calibrated by measuring the optical path loss, and the maximum error is only 1.2%. Finally, the reflectivity of the VBG measured by the calibrated system is 30.84%, which is basically consistent with the multi-physical field simulation results, showing a deviation as low as 0.85%. The experimental results fully verify the availability and high measurement accuracy of the reflectivity measurement system. This research work provides a new method for testing the characteristics of micron-scale grating size VBGs. Additionally, this work combines optical characterization methods to verify the good effect of VBG preparation technology, providing core technical support for the realization of subsequent homogeneous integrated Fabry–Perot cavity sensors. Furthermore, it holds important application value in the field of optical sensing and micro-nano integration.

## 1. Introduction

In the fields of real-time monitoring of aero-engine hot-end components [1], aerodynamic parameter capture of hypersonic vehicles [2], industrial process control and environmental monitoring [3], there are test requirements for high-precision in situ sensing in high-temperature environments exceeding 1000 K [4,5]. High fineness air-gap Fabry–Perot (F-P) cavity, as a new sensitive unit, can realize in situ detection of temperature [6], noise [7], pressure [8,9,10,11,12], and other parameters [13] by detecting small changes in air refractive index in the cavity. However, at present, the high fineness F-P cavity is mostly realized by the dielectric high-reflection film. The temperature resistance of the conventional dielectric high-reflection film is up to 300 °C. In the high-temperature environment of 1000 K, it is easy to crack and fall off due to the thermal stress mismatch, which results in the failure of the F-P cavity. Therefore, it is urgent to develop a high-reflection F-P cavity mirror element suitable for high-temperature in situ sensing.

As an emerging optical feedback element, reflective volume Bragg gratings (VBGs) are used to improve the wavelength stability and spectral performance of semiconductor lasers [14,15,16] due to their high reflectivity [17], narrow linewidth [18], and low scattering and absorption losses [19]. Based on these advantages, we propose to use the reflective VBG as the reflective cavity mirror of the air-gap F-P cavity to replace the traditional dielectric film mirror [20], and improve the temperature resistance while maintaining the high fineness of the F-P cavity. At present, the research on reflective VBG is mainly focused on the Photo-Thermo-Refractive (PTR) glass-based VBG [21]. The maximum working temperature of PTR glass is about 500 °C [22,23], which limits its application in a higher temperature environment. In contrast, the quartz-based VBG uses pure fused quartz material and can maintain structural stability when working at a high temperature of 1000 °C for a long time [24]. Moreover, the quartz-based VBG is prepared by forming a periodic permanent refractive index modification inside the material through femtosecond laser direct writing technology [25,26]. As a reflective cavity mirror of the quartz air-gap F-P cavity, it can effectively solve the problem of thermal stress mismatch and improve the high-temperature in situ sensing ability of the F-P cavity sensitive unit by virtue of the homogeneous material integrated structures.

The reflectivity of the VBG is the key parameter that is used as the F-P cavity mirror. The accurate measurement of the reflectivity plays an important supporting role in the sensing performance prediction of the F-P cavity [27,28,29,30]. Lauris Talbot et al. detected different reflection orders of VBGs by the transmission method. The broadband light of the supercontinuum light source is transmitted to the experimental device through a single-mode fiber. The core unit of the experimental device is a pair of calcium fluoride lenses, which can focus the light inside the VBG. After the transmitted light is refocused by a single-mode fiber, it is connected to a spectral analyzer for research [31]. Joelle Harb used a 1.55 μm single-frequency fiber laser to measure the diffraction efficiency of the VBG. The light beam from the fiber-coupled light source is collimated and focused inside the glass through a lens with a focal length of 40 cm. The transmitted light is refocused into the fiber connected to the spectrometer for testing [32]. Yan et al. built a VBG diffraction efficiency test device. The 1550 nm broadband light source is collimated by the optical fiber and vertically incident on the VBG. Then the outgoing light is imported into the spectrometer through the receiver for spectral analysis. The diffraction efficiency is calculated by the intensity of the transmission spectrum [33]. The transmission end of the reflectivity measurement system currently selected by the researchers receives the transmitted light through the optical fiber. The mismatch between the fiber end face and the grating area size of the VBG can easily cause the transmission light loss, resulting in certain measurement and calculation errors. In addition, the grating area size of the VBG measured by them is in the millimeter scale, and only a single lens is needed to focus the beam in the system. The system is not suitable for the reflectivity measurement of the VBG in the small grating area size (micron dimension).

In this paper, the preparation of a large depth ratio VBG is realized by a femtosecond laser multi-layer writing method. The consistency of processing is verified by microscopic imaging, SEM scanning, and 3D refractive index characterization. For the study of its reflection characteristics, a free-space optical path reflection measurement system is proposed. The ZEMAX (Ansys Zemax OpticStudio 2023 R1) simulation is used to simulate the polarization state transformation and transmission process of the space optical path, and the optimal position of each component is determined when the target spot size is realized. According to the system simulation results, a VBG reflectivity measurement system is built. The system is calibrated by obtaining the optical path loss and measurement error to verify its measurement accuracy. Finally, the reflectivity of the VBG measured by the calibrated system is 30.84%, and the deviation from the theoretical simulation value is 0.85%, which is within the measurement error range of the system calibration. On the one hand, the measurement results of VBG reflectivity verify the availability and accuracy of the measurement system. On the other hand, it shows that the consistency between the preparation of the VBG structure and the parameters of the simulation model is high, and the preparation effect is good. This study provides technical support for the use of VBGs instead of dielectric reflective films to achieve high-temperature and high fineness F-P cavities, which is of great significance in the field of micro-nano processing design for optical sensors.

## 2. Materials and Methods

### 2.1. Preparation and Characterization of Volume Bragg Grating

**Figure 1:**
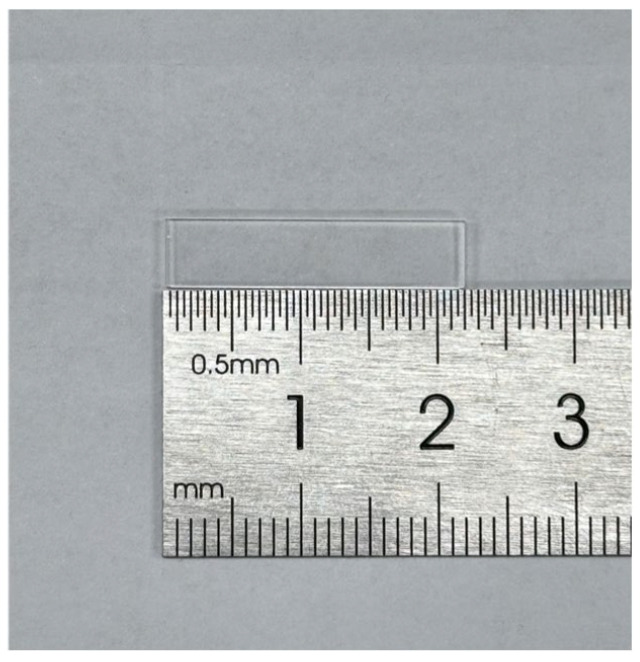
Quartz glass sample.

The substrate of the VBG is a 1 mm thick ultraviolet-grade fused quartz crystal block (JGS1). And the laser cuts it into a sample with a size of 22 × 4 × 1 mm^3^, as shown in Figure 1. The surface of the sample is mechanically polished to a surface roughness (Ra) of less than 0.5 nm and a surface warpage of less than 0.2°, which meets the accuracy requirements of femtosecond laser direct writing and optical microscope observation.

The VBG period is set to 1.609 μm. The glass sample is modified by a femtosecond laser direct writing system, of which the laser wavelength is 1030 nm and the maximum repetition frequency is 100 kHz. The repetition frequency directly affects the processing depth of VBG by regulating the thermal accumulation effect and the efficiency of pulse-material interaction. High repetition rate can provide higher pulse density at the same scanning speed, and heat accumulation can soften the surface layer of the material. Thus, the energy threshold of deep processing is reduced, and it is easier to achieve large depth processing. Therefore, 100 kHz is the best choice to ensure that the structure is not destroyed under the premise of high manufacturing efficiency. Further increasing the repetition rate will lead to significant thermal effects and structural damage. The beam is stretched to tens of microns using a spatial light modulator (SLM). The laser beam modified by SLM is projected onto the objective lens by a 4F system, and the beam diameter is reduced. Through the high numerical aperture objective lens (NA = 0.9), the femtosecond laser with an incident beam spot diameter of 4 mm is focused at a depth of 600 μm below the surface of the sample, and the energy density is higher than the material damage threshold. During the femtosecond laser direct writing process, the laser irradiation area undergoes a refractive index change. And each line in the grating is written only once; the line width of the single-layer grating is 200 nm.

**Figure 2:**
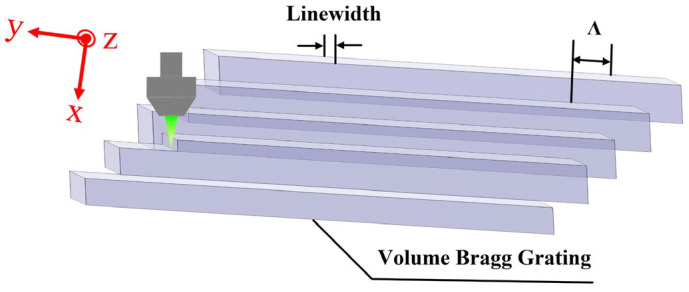
The preparation process schematic diagram of the VBG.

In order to increase the depth of VBG to improve its reflectivity, a multi-layer structure is formed by a single modified array through a multi-layer writing method. Each writing layer of the VBG partially overlaps with the next continuous layer, and it is repeated many times to construct a VBG structure with a large depth ratio. The specific implementation process is shown in Figure 2. Based on the large-range displacement stage, the femtosecond beam is focused at the bottom of the grating to write the first layer (x-axis) of the bottom layer (z-axis). The sample is translated along the y-axis and written directly to a length of 18 mm. Subsequently, the sample moves one cycle along the x-axis direction, and the focused beam directly writes the second layer (x-axis) of the bottom layer (z-axis). The process is repeated in turn to complete the writing of the bottom 1000 layers (x-axis). The sample is moved upward along the z-axis, repositioned to the position of the first layer for longitudinal splicing and writing, and the layer writing process is repeated. The sample continues to move upward along the z-axis and repeats the above process. Finally, a VBG with a large depth ratio is obtained, of which the longitudinal depth is up to 500 μm.

**Figure 3:**
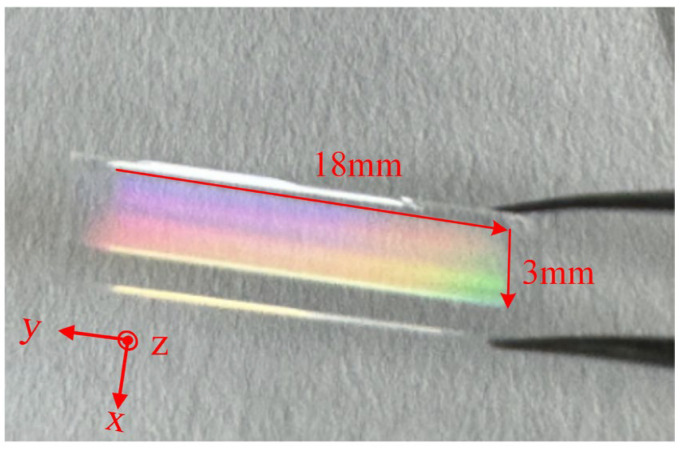
Photograph of the VBG diffracting ambient light. It has dimensions of 3 mm (X axis), 18 mm (Y axis), and 500 μm (Z axis).

**Figure 4:**
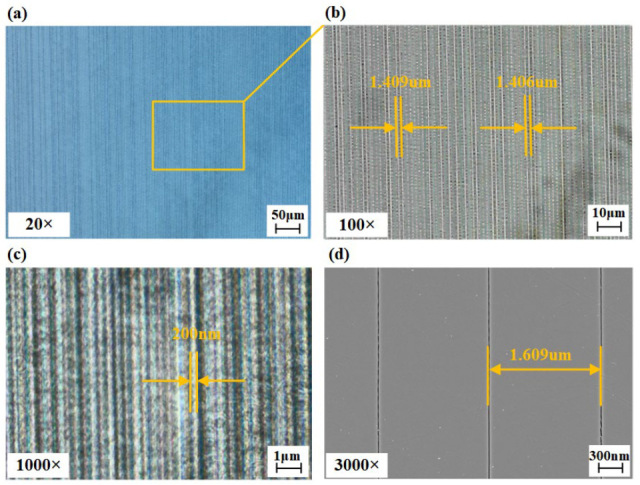
The characterization diagram of the VBG. (**a**) The schematic of the VBG structure under the 20× microscope. (**b**) The schematic of the VBG structure under the 100× microscope. (**c**) The schematic of the VBG structure under the 1000× microscope. (**d**) The schematic of the VBG structure under the 3000× scanning electron microscope.

The prepared VBG is shown in Figure 3. The size of the VBG region in the quartz glass sample is 18 mm × 3 mm. In order to better understand the morphology and processing quality of VBG, we use a variety of methods to characterize it. Firstly, the sample is placed under a high magnification microscope (Axio Imager 2, ZEISS, Oberkochen, Deutschland) and the morphology is observed by transmission light. The observation results of 20×, 100×, and 1000× are shown in Figure 4a–c. The selected VBG region is gradually amplified and tested. It can be clearly measured that the spacing between the two gratings is 1.408 μm, and the grating line width is 200 nm. After that, the field emission scanning electron microscope (SEM) is used to image and analyze the structure and morphology of the laser-modified area in detail. The sample is immersed in HF solution for 1 min, and the observed surface is coated with a gold layer. The volume grating structure can be clearly seen. The characterization results indicate that the prepared VBG has good cycle consistency, splicing continuity, and no obvious error.

**Figure 5:**
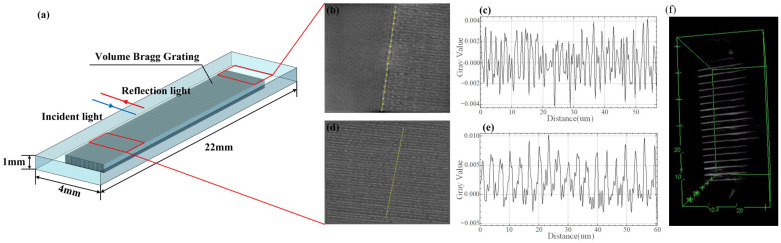
VBG refractive index test. (**a**) schematic diagram of sampling position. (**b**) initial laser inscription area. (**c**) distribution of refractive index difference at the initial position. (**d**) laser inscription intermediate area. (**e**) distribution of refractive index difference at the intermediate position. (**f**) refractive index characterization of 3D morphology (side view).

In order to further accurately measure the refractive index distribution and changes, a non-destructive and non-invasive 3D refractive index characterization imaging system is used. By combining optical imaging and image reconstruction technology, real-time observation and accurate analysis of the VBG structure are realized. In the experiment, 50× and 100× of the objective lens are used for observation. Increasing the lens multiple will make the morphology of the observed sample clear, but the field of view and brightness will be reduced accordingly. The precision displacement platform adjusts the distance, the CCD camera takes a sample above the sample, and the entire test instrument is controlled by the host computer system. Firstly, the blank substrate without grating is sampled, and then it is positioned in the VBG region for secondary sampling. Figure 5b,d are the VBG morphologies of different regions. It can be clearly seen from the two images that there is a light and dark contrast between the VBG region and the substrate region, that is, the comparison between the refractive index of the modified region and the refractive index of the substrate. Then, the refractive index change value is imaged and calculated by optical imaging and characterization technology. Through the comparative analysis of the control software, the refractive index change value of the yellow streak area is obtained. The yellow streak area in Figure 5b is the initial writing position of the laser. The laser energy is relatively concentrated, and the degree of modification is deep that the refractive index change is about 5.0 × 10^−3^. The yellow streak region in Figure 5d is the middle region of VBG, and the refractive index change is 1.0 × 10^−3^. It can be seen that the refractive index of the grating position is evenly distributed, and the period interval is uniform through the refractive index curve. It is once again confirmed that the written VBG has good period consistency. Finally, we sampled 300 sets of data and used optical imaging and image reconstruction techniques to reconstruct the three-dimensional morphology of the written VBG. As shown in Figure 5f, the effect of laser processing can be judged by the three-dimensional distribution information of the refractive index inside the VBG, which has a certain guiding effect on the optimization of processing parameters and deviations.

### 2.2. Reflection Characteristics Simulation of Volume Bragg Grating

**Figure 6:**
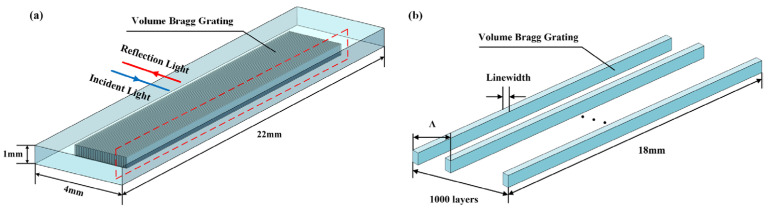
Structure diagram of the VBG. (**a**) Overall structure diagram. (**b**) Local structure enlarged diagram.

For the prepared VBG, the reflection characteristics are simulated by constructing the finite element model of the reflective VBG. Based on quartz glass, the geometric three-dimensional model of VBG is established. The size of the sample is set to be 22 mm × 4 mm × 1 mm, and the size of the single-layer VBG is 18 mm × 200 nm × 0.5 mm (where 18 mm and 0.5 mm are the grating length and grating depth, respectively, and 200 nm is the grating linewidth). The obtained three-dimensional geometry is shown in Figure 6. In the grating area, the free quadrilateral mesh is selected for meshing, and the rest of the mesh is automatically divided. In the simulation model, the grating period (Λ) is 1609 nm, the grating linewidth is 200 nm, the refractive index change is 1.0 × 10^−3^, and the number of grating layers is 1000 layers.

**Figure 7:**
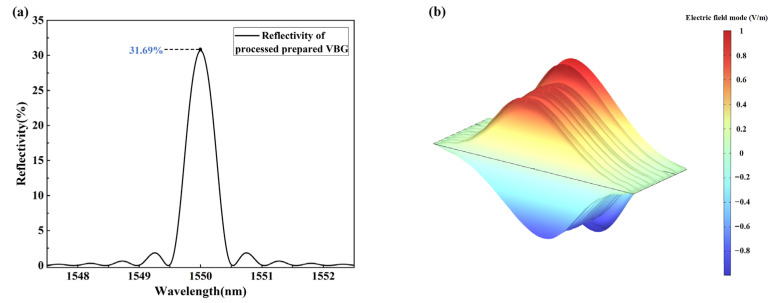
Simulation results of the VBG. (**a**) Theoretical reflectivity curve. (**b**) Electric field energy distribution.

The absorption and scattering loss of materials is not considered. When the light with a certain spectral width passes through the VBG, the light satisfying the grating equation condition is reflected back at the first layer of the grating, and the rest of the light continues to propagate forward. Part of the light is also reflected back at the second grating. The reflected light is continuously superimposed and can be coherently combined into a strong reflected light at the Bragg wavelength. According to the simulation model, the theoretical reflectivity of VBG is 31.69%. The reflection spectrum and electric field energy diagram are shown in Figure 7. It can be seen that the high electric field energy is mainly distributed in the grating region of the VBG.

### 2.3. Test System Design and Simulation

**Figure 8:**
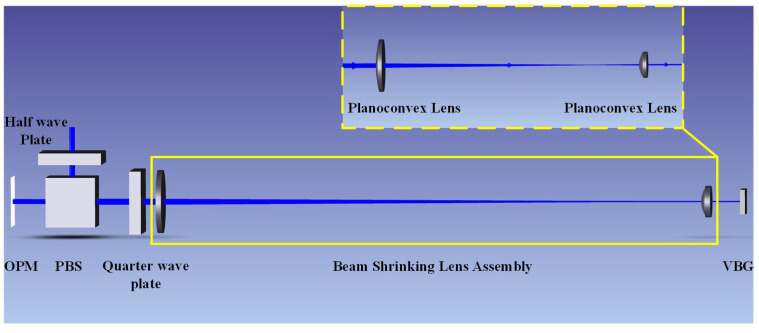
Simulated optical path diagram of the test system.

For the measurement of VBG reflectivity, a free-space optical path reflective measurement system is designed. In this system, the beam is normally incident on the VBG and then receives the light reflected back many times by the VBG. The reflected light returns in the original way in the space optical path. Considering that the reflection area of the VBG is relatively narrow (500 μm), it is necessary to design a beam-shrinking lens group with high imaging quality, precise collimation, and a stable beam reduction ratio to achieve the focusing of small light spots inside the VBG. Therefore, a Keplerian beam-shrinking lens group is selected. The selection of focal length parameters for the Keplerian beam-shrinking lens group needs to take into account the requirements of spot size and system stability. The size of the spot after beam reduction should be much smaller than the reflection surface width of the VBG to avoid energy loss caused by spot overflow and ensure the accuracy of the reflectivity measurement. At the same time, the lens spacing should be moderate to facilitate optical path debugging. Finally, the lens parameters of the Keplerian beam-shrinking lens group are determined as F1 = 100 mm and F2 = 20 mm. According to the beam reduction ratio = F2/F1, the incident spot can be theoretically reduced by five times. The system is simulated and optimized by ZEMAX optical design software(Ansys Zemax OpticStudio 2023 R1), and the complete simulation optical path is shown in Figure 8.

Based on the principle of polarization splitting, the polarization state of the beam is transformed to effectively detect the reflected light. The linearly polarized light emitted by the laser is used as the incident light. The polarization state of the incident light can be changed by rotating the angle of the half-wave plate. The polarization splitting prism (PBS) will select the transmitted polarization direction. The combination of the two can achieve continuous adjustment of light energy. Here, we use the reflected S-polarized light as the measurement beam incident on the measurement system. By rotating the quarter-wave plate, the beam is completely converted into circularly polarized light. So that when the beam is reflected back after passing through the VBG, it can be completely converted into P-polarized light after passing through the quarter-wave plate again, which is completely transmitted from the surface of the PBS splitting film. The selected beam-shrinking lens group is simulated using the sequential mode in ZEMAX, and the simulation results show that it can reduce the incident spot size by five times while maintaining collimated output. Finally, the devices are combined using the non-sequential mode to simulate the overall optical path effect. The simulation model fully verifies the type parameters and position distance of each device in the optical path under the ideal beam reduction effect and reflection effect, providing theoretical support for the construction of the actual measurement system.

## 3. Results and Discussion

### 3.1. Test System Construction

**Figure 9:**
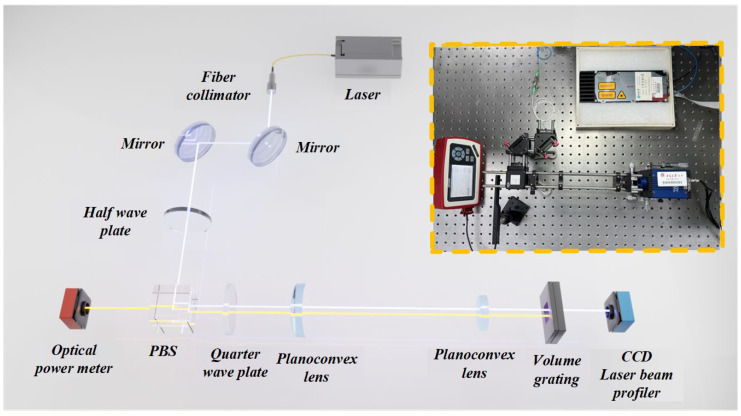
Physical diagram of the test system.

According to the simulation model, the spacing and focal length of each component in the test system are determined. The 30 mm coaxial system is used to improve the collimation of the optical path. Firstly, the super-continuous narrow-band laser (1550 nm, NKT E15) is used as the input beam, and then the half-wave plate, PBS, quarter-wave plate, and beam-shrinking lens group are successively fixed in the coaxial system, and the sample to be tested is fixed on the three-dimensional adjustment frame. The test system is built as shown in Figure 9. The optical path in the system is coarsely collimated by visible light. Then, the laser is used to hit the light passing through each component at a fixed central horizontal position through the cursor card. Whereafter, by slightly adjusting the position of the sample to be tested, the laser beam passing through the beam-shrinking lens group is perpendicular to it. Finally, after multiple calibrations, all components of the entire system are in a horizontal alignment position.

**Figure 10:**
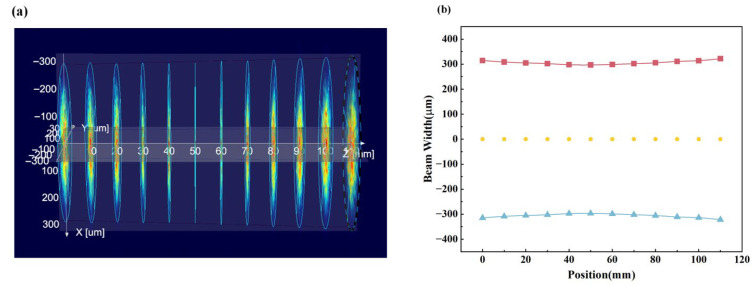
(**a**) The beam diameter measured by the beam quality analyzer. (**b**) The beam diameter fitting curve.

In particular, in order to improve the beam collimation and reduce the spot size of the laser output beam, the fiber collimator is connected to the polarization-maintaining optical output end of the laser. The working distance of the fiber collimator is 500 mm. The beam quality analyzer is used to characterize the beam properties of the fiber collimator to verify its working distance and beam waist radius. The beam waist position is the most concentrated and smallest place of the beam, which can make the best use of the characteristics of the optical elements to ensure the imaging quality. After that, the fiber collimator is placed in the coaxial system, and the beam is incident horizontally to the beam splitter and the quarter-wave plate through two 45° mirrors. The beam quality analyzer is placed behind the quarter-wave plate, and the position of the beam-shrinking lens group is accurately determined by testing the waist radius of the transmitted beam. The beam quality analyzer is moved along the beam propagation axis in the expected focal region, and the beam diameter and spot size within the propagation distance of 120 mm are recorded at an interval of 10 mm, as shown in Figure 10. At a distance of 45.05 mm from the quarter-wave plate, the first plano-convex lens is placed at the minimum point of the beam waist radius, that is, the minimum point of the spot. This placement can ensure that the convex lens is most effective in suppressing the divergence of the beam, reducing possible light loss and distortion, and improving the collimation and beam reduction efficiency of the system.

**Figure 11:**
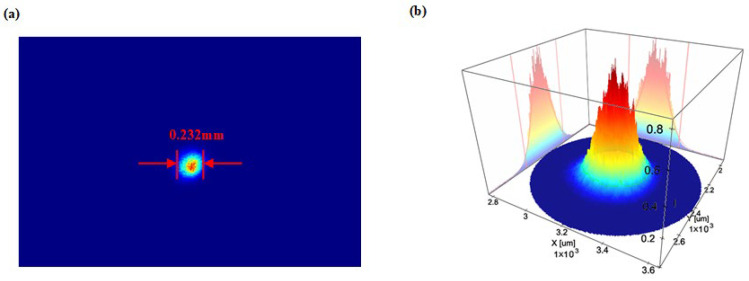
(**a**) The minimum spot size diameter. (**b**) spot energy distribution diagram.

When the spacing of the beam-shrinking lens group is adjusted, the beam quality analyzer is used to detect the size of the beam-shrinking spot in real time. After the adjustment, the output beam of the beam-shrinking lens group should be collimated, and the spot should be the smallest. The spot is scanned along the Y axis to find the ideal spot diameter position. As shown in Figure 11, the minimum spot diameter is about 0.232 mm. Furthermore, the beam parameter product (BPP) of the reduced beam is calculated as 1.1 mm·mrad, which is close to the minimum BPP that can be achieved by the diffraction-limited Gaussian beam. The above results fully show that the beam quality of the reduced beam is great. In the free space optical path reflective measurement system, the VBG is placed at the measured minimum spot diameter, of which the reflectivity can be detected by an optical power meter at the left end of the PBS.

### 3.2. Test System Calibration

**Figure 12:**
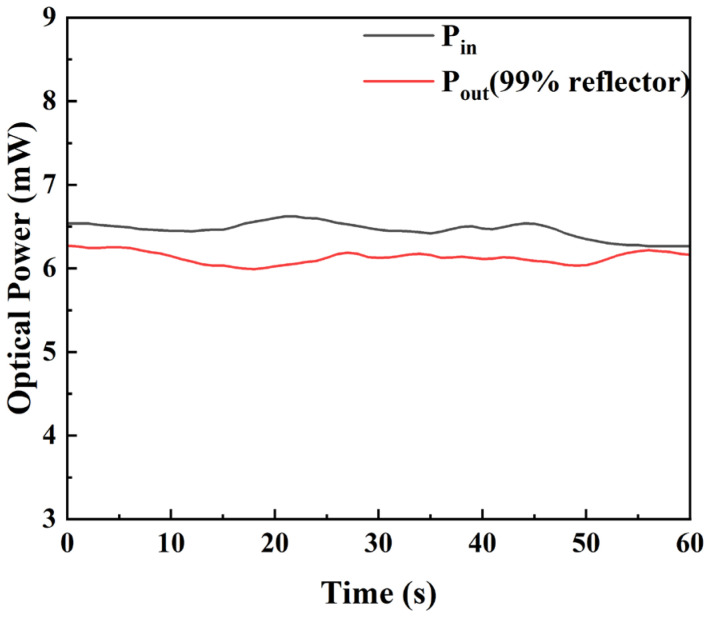
The fluctuation schematic diagram of the incident and the reflected light power of the test system with time.

In order to accurately measure the reflectivity of the VBG, the testing accuracy of the system is verified by the calibration of the transmission loss and measurement error of the free space optical path reflective measurement system. Firstly, the transmission loss of the optical path system is measured by using a commercial plane high reflector (reflectivity ≥99%, Thorlabs). The planar high reflector is placed in the same position as the VBG, and the optical power meter (OPM, Thorlabs, Newton, NJ, USA) is used to detect the incident light power and the reflected light power of the space system, respectively. Before the test, the display value of the OPM is cleared to avoid the influence of background stray light. The optical power meter probe is placed on the right side of the PBS as the incident light power detection position. The angle of the half-wave plate is rotated to convert the polarization state of the incident polarized light. The optical power is recorded in real time during the rotation process. The maximum optical power is recorded as the calibration incident light power *P_in_*′, and the position of the half-wave plate is fixed. The probe of the OPM is removed, so that the laser passes through a quarter-wave plate and a beam-shrinking lens group, incident to the end face of the plane high reflector. The optical power meter probe is placed on the left side of the PBS as the reflected light power detection position. By rotating the angle of the quarter-wave plate, the incident linearly polarized light is transformed into completely circularly polarized light after passing through the quarter-wave plate. And the reflected light reflected by the plane high reflector is transformed into a single polarized light after passing through the quarter-wave plate again. The maximum optical power at the detection position of the reflected light power is recorded as the reflected light power *P_out_*′.

(1) $$L=1-{\frac{P_{out}\prime }{P_{in}\prime \times R}}_{0}\times 100\%$$

where *L* is the inherent optical path loss of the experimental system, and *R*_0_ is the reflectivity of the plane high reflector. The change in incident power and output power with time is detected as shown in Figure 12. The average incident power is 6.463 mW, and the average outgoing power is 6.128 mW. The optical path loss of the test system can be calculated by Equation (1) to be 4.23%. Based on this loss value, subsequent tests will be performed.

Based on the transmission loss of the system, the measurement error of the system is calibrated by measuring the reflectivity of various reflectors, including dielectric film reflectors with 30%, 50%, and 99.9% reflectivity, and silicon wafers with 50.4% reflectivity. The measured data are shown in the table, where the measured reflectivity is derived from Equation (1). From the multiple measurement results as shown in Table 1, the maximum measurement error of the system is 1.2%. The deviation between the experimentally measured values and the theoretical values mainly stems from the inherent optical path loss of the experimental system and laboratory environmental factors, including the installation angle deviation of reflective devices and the optical path alignment fluctuation caused by slight vibrations in the laboratory.

### 3.3. Reflection Characteristics Test of VBG

The calibrated test system is used to test the reflectivity of the VBG. It is known that the overall optical path loss is 4.23%. By replacing the standard reflector in the test system with a VBG, the light is vertically incident on the VBG area to be tested. Before the test, the display value of the OPM is cleared to avoid the influence of background stray light. Firstly, the OPM probe is placed between the PBS and the quarter-wave plate as the first position *P_in_*. Then, the OPM probe is placed on the left side of the PBS, which is recorded as the second position *P_out_* to measure the light reflected by the VBG to be tested. Based on the measured optical power, the reflectivity of the VBG is 30.59% according to Equation (1). And the measurement deviation is 0.85% compared with the theoretical reflectivity, which is within the maximum measurement error range of the system calibration. The measurement results of VBG reflectivity are shown in Table 2, which again verify the accuracy of the measurement system. At the same time, it also exhibits the good preparation effect of the VBG, which is in great agreement with the simulation model.

## 4. Conclusions

In summary, this study proposes a free-space optical path reflective measurement system for the reflectivity of quartz-based VBGs, which serves as a critical foundation and prerequisite for subsequent application-oriented research on quartz-based VBGs. Through ZEMAX optimization simulation and error calibration using standard reflectors, the measured reflectivity of the quartz-based VBG is 30.84%, with a deviation of only 0.85% from the predicted value of the multi-physical field theory. This result fully verifies the accuracy of the measurement system and, meanwhile, provides a new and reliable method for testing the reflection characteristics of VBGs with micron-scale grating regions. Notably, this study is highly aligned with the application direction of quartz-based VBGs in high-temperature micro F-P cavity sensing elements. The inherent advantages of quartz-based VBGs, including excellent thermal stability, compact size, and micro-integration compatibility, are exactly the core strengths urgently required for this application scenario. To verify the feasibility of this application and build a bridge between basic characterization and practical application, this study has initially conducted preliminary experiments on the reflectivity variation characteristics of the VBG under different temperatures. The preliminary results show that the reflectivity of the VBG remains stable with a variation amplitude of less than 2% within the temperature range from room temperature to 700 °C. When the temperature exceeds 700 °C, the rate of reflectivity attenuation accelerates, and specifically, the reflectivity decreases by approximately 6% in the temperature segment from 700 °C to 800 °C. This variation trend not only clarifies the initial high-temperature performance boundary of the VBG but also provides data support for subsequent optimization work (e.g., exploring high-temperature annealing pretreatment to improve thermal stability). Furthermore, this study lays a necessary foundation for promoting the application of VBGs in the field of high-temperature in situ sensing based on F-P cavities.

## Figures and Tables

**Table 1:** Reflectivity measurement data of various reflectors.

Type	Standard Reflectivity	Average Power of Incident Light	Average Power of Reflected Light	Tested Reflectivity	Measurement Error
Dielectric film reflector 1	30%	6.463	1.783	28.8%	1.2%
Dielectric film reflector 2	50%	6.463	3.045	49.2%	0.8%
Dielectric film reflector 3	99.9%	6.463	6.122	98.9%	1.0%
Silicon wafer	50.4%	6.463	3.051	49.3%	0.9%

**Table 2:** VBG reflectivity measurement data.

Type	Theoretical Reflectivity	Average Power of Incident Light	Average Power of Reflected Light	Tested Reflectivity	Measurement Error
VBG	31.69%	6.463	1.909	30.84%	0.85%

## Data Availability

The data that support the findings of this study are available from the corresponding author upon reasonable request.

## References

[1] Zhang Y., Jiang Y., Yang S., Zhang D. (2024). All-sapphire fiber-optic sensor for the simultaneous measurement of ultra-high temperature and high pressure. Opt. Express.

[2] Zhao S., Jiang Y., Zhang Y., Deng H. (2025). High-Sensitivity Sapphire Vacuum Fiber Pressure Sensor for High Temperature Applications. IEEE Sens. J..

[3] Deng Y., Jiang J. (2022). Optical Fiber Sensors in Extreme Temperature and Radiation Environments: A Review. IEEE Sens. J..

[4] Ma S., Xu Y., Pang Y., Zhao X., Li Y., Qin Z., Liu Z., Lu P., Bao X. (2022). Optical Fiber Sensors for High-Temperature Monitoring: A Review. Sensors.

[5] Zhang Y., Jiang Y., Deng H., Gao H., Tang C., Wang X. (2023). All-sapphire-based optical fiber pressure sensor with an ultra-wide pressure range based on femtosecond laser micromachining and direct bonding. Opt. Express.

[6] Gao S., Liu Y., Yang J., Bai Y., Chen Y., Shi J., Yuan L., Guan C. (2025). Ultracompact Fabry-Perot interferometer based on femtosecond laser-assisted wet etching for high-temperature sensing. Opt. Express.

[7] Xi Q., Ma B., Tian Z., Li R., Wang Y., Ma Z. (2025). Micro-Fabricated Compact Extrinsic Fabry-Perot Sensor for In-Situ Harsh Environment Acoustic Measurement. IEEE Photonics J..

[8] Zhao X., Ren X., Bai J., Yang Y., Chen J., Zheng Y., Xue C. (2025). A High-Sensitivity and High-Temperature-Resistant Gas Pressure Sensor Based on Open Hollow-Core Fibers Fabry–Pérot Interferometer Probe. J. Light. Technol..

[9] Shao Z., Chen M., Shan Z., Sun Z., Wang Y., Yan L., Wang Y., Liu B. (2024). High Sensitivity All Sapphire-Based Optical Fiber Fabry–Perot Pressure Sensor for Harsh Environment. IEEE Trans. Instrum. Meas..

[10] Wang S., Wang J., Li W., Liu Y., Li J., Jia P. (2022). A MEMS-Based High-Fineness Fiber-Optic Fabry–Perot Pressure Sensor for High-Temperature Application. Micromachines.

[11] Liao Y., Liu J., Dai Y., Wang J., Wan S., Zhang L., Wang H., Jia P. (2025). Temperature-compensated fiber-optic Fabry-Perot pressure sensor based on sapphire MEMS technology for high temperature environment up to 1500 °C. Opt. Express.

[12] Chen Y., Yang P., Wang G. (2025). High-Sensitive Fiber-Optic Gas Pressure Sensor Consisting of Cascaded Polymer–Air Cavities Based on Vernier Effect. IEEE Sens. J..

[13] Birri A., Sweeney D.C., Hyer H.C., Schreiber B., Cakmak E., Petrie C.M. (2025). A Miniaturized, High-Bandwidth Optical Fiber Fabry–Perot Cavity Vibration Sensor Demonstrated up to 800 °C. IEEE Sens. J..

[14] Liu Y., Liu Y., Li H., Xiao H., Xia Y., Gao R., Li X., Zheng Q. (2022). Wavelength stabilization and spectra narrowing of a 405 nm external-cavity semiconductor laser based on a volume Bragg grating. Appl. Opt..

[15] Li R., Huang S., Nie H., Zhang J., Ma T., Zhang J., Chen Z., Jia C., Yao B., Xia J. (2025). High power narrow bandwidth picosecond PPLN-OPO. Opt. Express.

[16] Han J., Zhang J., Zhang Y., Peng H., Zhang J., Ye S., Shan X., Wang L. (2025). Tunable narrow linewidth diode laser based on a fibre-coupled external cavity feedback structure. Opt. Laser Technol..

[17] Wang M., Yan C., Liu X., Sun S., Qiu J. (2025). Efficient fabrication of volume diffraction gratings in sapphire with a femtosecond laser. Mater. Lett..

[18] Chai C., Yan Y., Cao B., Guo L., Huang L., Xu S., Zhao S. (2025). Low melting temperature chloride borosilicate photothermal refractive glass for holographic applications. Ceram. Int..

[19] Fagginger Auer F.J., Keller C.U. (2024). Stacking spectral lines with multiplexed Bragg gratings in an acousto-optical tunable filter. Opt. Express.

[20] Wang H., Chen J., Wen F., Zheng Z., Han H., Zheng Y., Xue C. (2025). Volume Bragg grating with tunable reflectivity and the bandwidth assisted Fabry-Pérot cavity. Opt. Express.

[21] Ge Z., Xiong B., Li Q., Zhang X., Yuan X. (2024). The effect of photo-thermo-induced crystallization on the properties and microstructure of transmission volume Bragg gratings. Ceram. Int..

[22] Ge Z., Xiong B., Zhang X., Yuan X. (2024). Evolution of microstructure and spectral characteristics of silver nanoparticles in photo-thermo-refractive glass. J. Non-Cryst. Solids.

[23] Ge Z., Xiong B., Mo D., Chen X., Zhang X., Yuan X. (2023). Crystallization heat treatments for the fabrication of volume Bragg gratings based on photo-thermo-refractive glass. Opt. Mater..

[24] Yao H., Pugliese D., Lancry M., Dai Y. (2024). Ultrafast Laser Direct Writing Nanogratings and their Engineering in Transparent Materials. Laser Photonics Rev..

[25] Talbot L., Per Siems M., Richter D., David N., Blais-Ouellette S., Nolte S., Bernier M. (2024). Mid-infrared tunable filter based on a femtosecond-written silica volume Bragg grating. Opt. Lett..

[26] Ren G., Sun H., Nakagawa K., Sugita N., Ito Y. (2024). Crackless high-aspect-ratio processing of a silica glass with a temporally shaped ultrafast laser. Opt. Lett..

[27] Fang X., Cui C., Zhang K., Chen X., Pan S. (2025). High-power narrow-linewidth nanosecond Ti:sapphire laser with volume Bragg grating. Opt. Express.

[28] Talbot L., Bernier M. (2023). Femtosecond writing of intra-phase-mask volume Bragg gratings. Opt. Lett..

[29] Richter D., Siems M.P., Middents W.J., Heck M., Goebel T.A., Matzdorf C., Krämer R.G., Tünnermann A., Nolte S. (2017). Minimizing residual spectral drift in laser diode bars using femtosecond-written volume Bragg gratings in fused silica. Opt. Lett..

[30] He J., Zhao S.L., Guo L.W., Hua Y.J., Ye R.G., Xu S.Q. (2024). Design of reflective volume Bragg grating and its applicationin lasers. J. Optoelectron. Laser.

[31] Talbot L., Richter D., Heck M., Nolte S., Bernier M. (2020). Femtosecond-written volume Bragg gratings in fluoride glasses. Opt. Lett..

[32] Harb J., Talbot L., Petit Y., Bernier M., Canioni L. (2023). Demonstration of Type A volume Bragg gratings inscribed with a femtosecond Gaussian-Bessel laser beam. Opt. Express.

[33] Yan Y.H., Chai C.P., Guo L.W., Hua Y.J., Xu S.Q., Zhao S.L. (2024). Preparation and Performance of Holographic Grating Based on Novel Chlorine-Containing Photothermal Refractive Glass. J. Chin. Ceram. Soc..

